# Three-Phase Confusion Learning

**DOI:** 10.3390/e27020199

**Published:** 2025-02-14

**Authors:** Filippo Caleca, Simone Tibaldi, Elisa Ercolessi

**Affiliations:** 1 Laboratoire de Physique, Centre Nationale de la Recherche Scientifique, École Normale Supérieure de Lyon, Université Lyon 1, 46 Allée d’Italie, F-69342 Lyon, France; 2 Dipartimento di Fisica e Astronomia, Università di Bologna, Via Irnerio 46, I-40127 Bologna, Italy; simone.tibaldi2@unibo.it (S.T.); elisa.ercolessi@unibo.it (E.E.); 3 Stituto Nazionale di Fisica Nucleare, Sezione di Bologna, I-40127 Bologna, Italy

**Keywords:** neural networks, quantum many-body physics, condensed matter

## Abstract

The use of Neural Networks in quantum many-body theory has undergone a formidable rise in recent years. Among the many possible applications, their pattern recognition power can be utilized when dealing with the study of equilibrium phase diagrams. Learning by Confusion has emerged as an interesting and unbiased scheme within this context. This technique involves systematically reassigning labels to the data in various ways, followed by training and testing the Neural Network. While random labeling results in low accuracy, the method reveals a peak in accuracy when the data are correctly and meaningfully partitioned, even if the correct labeling is initially unknown. Here, we propose a generalization of this confusion scheme for systems with more than two phases, for which it was originally proposed. Our construction relies on the use of a slightly different Neural Network: from a binary classifier, we move to a ternary one, which is more suitable to detect systems exhibiting three phases. After introducing this construction, we test it on free and interacting Kitaev chains and on the one-dimensional Extended Hubbard model, consistently achieving results that are compatible with previous works. Our work opens the way to wider use of Learning by Confusion, demonstrating once more the usefulness of Machine Learning to address quantum many-body problems.

## 1. Introduction

The exponential growth in computational resources necessary to solve the quantum many-body problem often restricts access to exact solutions, with some notable exceptions [1]. Consequently, numerical techniques are frequently employed to validate physical intuitions or make predictions. Among these techniques are Quantum Monte Carlo [2], Density Matrix Renormalization Group (DMRG) [3], and Variational Monte Carlo [4]. Recently, especially in phase diagram reconstruction, Machine Learning algorithms have proven valuable in confirming theoretical data [5,6] and even suggesting unexplored phases [7,8,9,10,11,12,13,14].

Within this context, Learning by Confusion [15] has emerged as an interesting unsupervised technique that uses Neural Networks to find phase transition points in quantum many-body systems. It is based on the idea that a peak in accuracy is found corresponding to a phase transition when one trains a Neural Network multiple times with the same dataset labeled differently. Indeed, random labeling tends to result in lower accuracy as the network learns from incorrectly assigned labels, whereas unknown accurate labeling is more likely to yield high accuracies. This is the signal that we have found an optimal means of separating the data, and, in our context, we have found a phase transition. Learning by Confusion was first tested to detect the topological transition in the free Kitaev chain, the thermal phase transition in the classical Ising model, and the many-body localization transition in the random-field Heisenberg chain [15]. Later, it was also used successfully to detect first-order phase transitions [16], transitions in frustrated magnetic models [17], nuclear liquid–gas transitions [18], and entanglement breakdown [19].

This method was demonstrated to be efficient at detecting phase transitions in an unsupervised way when the dataset represents two different phases, and the original scheme was also employed to study some models with multiple phase transitions [20,21,22]. In this paper, we consider different models that display three phases separated by two different phase transition points where the standard technique fails to achieve the desired accuracy in distinguishing the two points. We propose a generalization of the Learning by Confusion formalism that we refer to as three-phase learning, while we denote the standard technique as two-phase learning. Our method is based on the possibility of studying a region of the phase diagram displaying three different phases via ternary, instead of binary, labeling.

The paper is structured as follows. In Section 2, we explain in detail how two-phase and three-phase learning work. In Section 3, we apply two-phase learning and our new method, three-phase learning, to four non-trivial models in quantum many-body physics: the Kitaev chain, in its free and interacting versions, and the one-dimensional Extended Hubbard model with two different shoulder potentials. Finally, in Section 4, we draw the main conclusions and possible outlooks of this work.

## 2. Confusion Learning

Learning by Confusion is a widely known and successful unsupervised method to determine phase transition points [15,23,24]. The main idea is to use a Neural Network to determine the phase transition point of a quantum system by scrambling the dataset until a good performance is reached. The data used in our case are composed of observables (which should signal the presence of a phase transition) computed for different phase diagram points and defined in Appendix A. Each data point consists of multiple observables arranged into a matrix form. Therefore, the network we consider in this work is a Convolutional Neural Network (CNN), which is widely used in pattern recognition problems [25]. A full description of the structure and functioning of CNNs is beyond our scope, and we direct the interested reader to [26,27,28] and Appendix A for further details.

### 2.1. Two-Phase Learning

**Figure 1:**
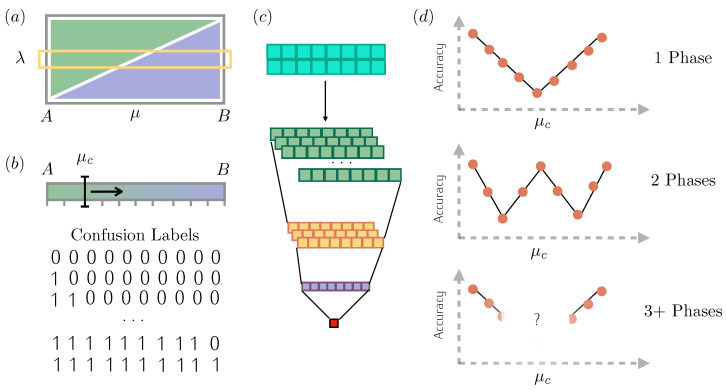
**Confusion learning**. (**a**) We start by selecting a line of the phase diagram that may or may not cross a phase transition by fixing one parameter and changing the other one (in this example phase diagram, λ is fixed and μ is changed). (**b**) By sweeping a parameter $\mu_{c}$ in the discretized interval [A,B], we generate different labeling for our data, going from all zeros to all ones. (**c**) Scheme of the Convolutional Neural Network used in the process. Blue represents the input data; green, yellow, and purple indicate the intermediate layers; and, finally, the accuracy is read from the red square representing the output neuron. For each labeling, we train a Convolutional Neural Network and plot its accuracy. (**d**) We expect the canonical *V*-shape or *W*-shape in the case of no (**top panel**) or one (**middle panel**) phase transition, while the outcome in the presence of three or more phases is unknown, as shown in the (**lower panel**).

To provide intuition regarding the algorithm, imagine a dataset of points that depends only on one parameter, e.g., μ, with the dataset generated by sweeping the parameter μ within an interval [A,B], or by varying one of the parameters while keeping the others fixed in the case of multiple parameters, as shown in Figure 1a. Every element of the dataset (data point) represents the values of the chosen observables, which are arranged to form a matrix. Then, it is assigned to a label, “0” or “1”, and the CNN is then trained to learn how to assign labels correctly. The degree of precision achieved by the network is evaluated by computing the *accuracy*, which is defined as the ratio between the number of right guesses and the total number of guesses over a test set. It is important to stress that data belonging to the test set are not fed to the network during the learning process.

In Learning by Confusion, we select an interval [A,B] of discrete points and a sweeping parameter $\mu_{c}$. Initially, we set $\mu_{c}=A$ and we label the dataset uniformly, e.g., assign to each of the discrete points in the interval the label “0” corresponding to one of either phase (Figure 1b). The CNN is subsequently trained and tested (Figure 1c). As all points are assigned the same label, the network learns easily to associate the label “0” with every input, resulting in a perfect accuracy of 1. Once that has been completed, we set $\mu_{c}$ to the second element of the interval, and the dataset is relabeled: the first element of the dataset is now assigned the label “1”, while the rest of the data points remain “0”. The CNN is then trained, validated, and tested again. Now, the accuracy is expected to decrease because we are forcing the CNN to classify the dataset incorrectly. We then proceed to relabel the data by assigning the first two data points to “1” and the following ones to “0”. These steps represent the confusion part of the algorithm because we are deliberately mislabeling our dataset. The process is repeated until uniform “1” labeling is achieved, and therefore again perfect accuracy is reached. We call this process 2-phase Learning by Confusion.

By plotting the accuracies obtained, we encounter three possible scenarios. If the portion of the phase diagram belongs to the same phase, we will see a characteristic V-shape, with the lowest accuracy of 50% being reached in the middle of the interval swept, as shown in the top panel of Figure 1d.

If the system undergoes a phase transition in the phase diagram region swept by the dataset, at a certain point during the confusion process, the data will be correctly labeled according to the two phases. If that is the case, we expect a peak in accuracy because the dataset is now labeled in a sensible way that the CNN can understand. This results in an *accuracy function* characterized by the so-called W-shape of the accuracy plot [15]. This is shown in the middle panel of Figure 1d.

Finally, if there is more than one phase transition, it is not easy to predict the behavior of the accuracy function (lower panel of Figure 1d).

### 2.2. Three-Phase Learning

Building on the 2-phase learning technique, we considered an intuitive yet unexplored extension of the model to detect two different phase transition points. In this case, consider two possible transition values, $\mu_{c}^{\left(1\right)},\;\mu_{c}^{\left(2\right)}$, which are to be found by sweeping both of them through the discretized interval [A,B]. At each step, the data points, which correspond to possible values of the coupling constant μ, are assigned a ternary label, chosen among “0”, “1”, and “2”, respectively, for the three cases: $\mu \leq \mu_{c}^{\left(1\right)}$, $\mu_{c}^{\left(1\right)}<\mu \leq \mu_{c}^{\left(2\right)}$, $\mu >\mu_{c}^{\left(2\right)}$ (here, we have assumed that $\mu_{c}^{\left(1\right)}<\;\mu_{c}^{\left(2\right)}$ (a similar procedure can be used for $\mu_{c}^{\left(1\right)}>\;\mu_{c}^{\left(2\right)}$). This operation results in an accuracy matrix, with columns/rows labeled by different values for the first/second transition parameters $\mu_{c}^{\left(1\right)}$, $\mu_{c}^{\left(2\right)}$ respectively. Each entry represents the accuracy obtained by training the CNN as just explained. In this way, we obtain an accuracy that depends on two variables, which we graphically represent via a contour plot. The plot is expected to display large accuracy values at the vertex points, corresponding to the learning of the trivial uniform assignments of the “0”, “1”, and “2” labels. In addition to that, a maximum is expected in the interior of the graph for the values of ($\mu_{c}^{\left(1\right)}$, $\mu_{c}^{\left(2\right)}$) coinciding with the two phase transition points. The results are symmetric with respect to the diagonal as the transposition operation simply corresponds to an inversion between labels “1” and “2”.

It must be stressed that, contrary to naive intuition, fixing one phase transition point, e.g., $\mu_{c}^{\left(1\right)}$, while sweeping the second one is not equivalent to performing 2-phase learning on a reduced dataset. One should keep in mind that the underlying CNN has been modified from a binary classifier to a ternary one; this naturally has non-trivial consequences on the output.

## 3. Results

We summarize the results of applying two-phase and three-phase learning to sections of the phase diagrams of four models: the Kitaev chain in its normal and interacting forms and the one-dimensional Extended Hubbard model with two different interaction ranges and particle fillings. For each model, we briefly present its phase diagram and the results obtained with the confusion scheme, leaving the details of the data and implementation to Appendix A. Unlike in [15], we do not calculate the entanglement spectra of the models but rather the correlator functions, as detailed below. We follow a previous work [5] in which, motivated by the fact that correlation functions represent data that could be obtained by experimental measurements, it was shown that the correlators are very effective at detecting phase transitions with different ML methods (see also Ref. [6]). Moreover, they can be obtained numerically in many ways, in contrast to the entanglement spectrum, which is primarily studied with DMRG techniques.

### 3.1. Kitaev Chain

#### 3.1.1. Free Model

The one-dimensional Kitaev chain [29] is a pedagogical model to show superconducting and topological effects. Given a chain of *L* sites, the Hamiltonian of the non–interacting (NI) version can be written as

(1) $$H^{K}=\sum_{i=1}^{L}\left(Ja_{i}^{†}a_{i+1}+\Delta \;a_{i}a_{i+1}+h.c.\right)+\mu \sum_{i=1}^{L}a_{i}^{†}a_{i}.$$

Here, $a_{i}^{†}\;\left(a_{i}\right)$ creates (annihilates) a spinless fermion on site *i*, *J* is the nearest neighbor hopping coefficient, Δ is the superconducting pairing, and μ is the chemical potential. We consider Periodic Boundary Conditions (PBCs); i.e., we set $a_{L+1}\equiv a_{1}$. Both in this model and the next one, we set L=100, but we noticed no relevant changes considering a larger size of L=200 sites. By going to momentum space and performing a Bogoliubov transformation, we can cast $H^{K}$ into a diagonal form $H^{K}=\sum_{k}E\left(k\right)\eta_{k}^{†}\eta_{k},$ where $\eta_{k}$ are Bogoliubov operators and the single-particle energy E(k) is provided by

(2) $$E\left(k\right)=2\sqrt{h_{z}{\left(k\right)}^{2}+h_{y}{\left(k\right)}^{2}},$$

with

(3) $$\begin{array}{c} h_{z}\left(k\right)=J\cos k+\mu /2,\;h_{y}\left(k\right)=\Delta \sin k. \end{array}$$

**Figure 2:**
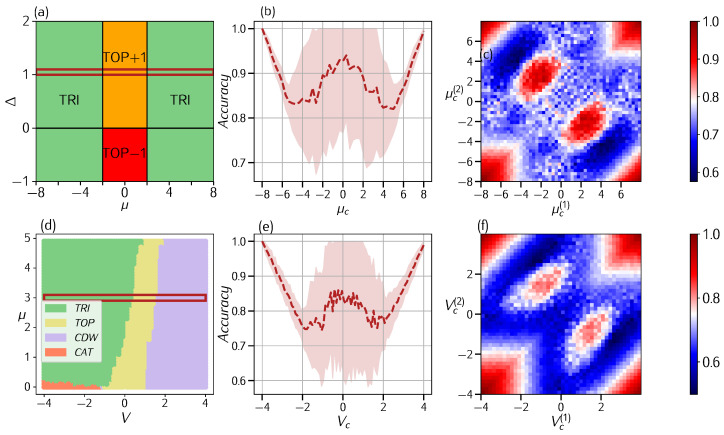
**Two-phase and three-phase learning on the Kitaev model**. The free Kitaev model: (**a**) phase diagram for μ∈[−8,8],Δ∈[−1,2], presenting one trivial phase (TRI) and two topological phases (TOP+1/TOP−1) for |μ|≤2Δ. In red, at Δ=1, the line chosen to test the models. (**b**) 2-phase learning applied to Kitaev. (**c**) 3-phase learning that predicts the two phase transitions at $\left(\mu_{c}^{\left(1\right)},\mu_{c}^{\left(2\right)}\right)=\left(1.92,-1.92\right)$. Interacting Kitaev model: (**d**) phase diagram; in red is the section considered for confusion learning at μ=3. (**e**) 2-phase learning shows inconclusive results. (**f**) 3-phase learning shows a peak at two phase transition points, $V_{c}^{\left(1\right)},V_{c}^{\left(2\right)}$.

This model describes a one-dimensional topological superconductor belonging to the BDI symmetry class [30,31,32] according to the ten-fold way [33], meaning that it admits time-reversal, particle–hole, and chiral symmetry. In this symmetry class, each topological phase is identified by a non-trivial *winding number*. The phase diagram is shown in Figure 2: two topological phases are present for |μ|<2Δ, with winding number ±1 (named TOP+1 and TOP−1); the only other phase is trivial (TRI).

We test two-phase learning, choosing Δ=1 and varying our trial transition point $\mu_{c}\in \left[-8,8\right]$. The accuracy obtained is shown in Figure 2b, and it does not respect the *W* shape, highlighting the possibility of two phase transitions. Therefore, we apply three-phase learning with the two trial transition points $\mu_{c}^{\left(1\right)},\mu_{c}^{\left(2\right)}$ varying in the same range and obtain the accuracy matrix whose contour plot is shown in Figure 2c. A peak in accuracy, which is obtained for two very different values of the chemical potential, can be clearly observed. The actual numerical data provide $\left(\mu_{c}^{\left(1\right)},\mu_{c}^{\left(2\right)}\right)∼\left(1.92,-1.92\right)$, values that are relatively close the analytical values, namely $\mu_{c}=\pm 2$.

#### 3.1.2. Interacting Model

Adding an interaction term, we obtain the interacting Kitaev chain, already studied in [34,35,36,37,38,39]

(4) $$H_{\mathrm{int}}^{K}=H^{K}+V\sum_{i}n_{i}n_{i+1}$$

where $n_{i}=a_{i}^{†}a_{i}$ is the occupation number at site *i*. This model cannot be solved exactly due to the interacting potential. We have reproduced the phase diagram, shown in Figure 2d, using the DMRG algorithm [40] with the ITensor package [41] after setting J=Δ=1. The topological phase (TOP, yellow) was detected through the presence of a Majorana edge mode in the ground state with open boundary conditions obtained via DMRG. The other phases are [35,36,37,38] a so-called Schrodinger’s cat-like phase (CAT, orange), a Charge Density Wave phase (CDW, purple), and a trivial phase (TRI, light green).

We applied the confusion scheme to the line at μ=3 with varying *V*. The plot in Figure 2b shows the accuracy obtained by varying the transition point $V_{c}$ in the range [−4,4]. Since there is not only one phase transition, we do not obtain the expected W-shape. Once again, the three-phase learning plot shows one peak in the interior of the square, with its coordinates corresponding to the two phase transition points. Although it is not precise, it predicts two phase transitions at $\left(V_{c}^{\left(1\right)},V_{c}^{\left(2\right)}\right)∼\left(-1,1.5\right)$.

### 3.2. Extended Hubbard

The fermionic Hubbard model [42,43] has been thoroughly studied in the past due to its solvability in one dimension, which can provide physical insights regarding strongly correlated electronic systems, and due to the growing possibilities of simulating it through quantum technologies [44,45,46]. In recent years, extensions of this model have been studied to investigate high-temperature superconductivity [47,48].

In the following, we will consider the one-dimensional Hamiltonian [49]

(5) $$H=-t\sum_{i,\sigma =↑,↓}\left(a_{i+1,\sigma }^{†}\;a_{i,\sigma }+h.c.\right)+U\sum_{i}n_{i↑}n_{i↓}+V\sum_{i}\sum_{l=1}^{r_{c}}n_{i}n_{i+l}$$

where $a_{i,\sigma }^{†}$, $a_{i,\sigma }$ are the usual fermionic creation and annihilation operators for particles of spin σ=↑,↓, $n_{i\sigma }=a_{i,\sigma }^{†}a_{i,\sigma }$ and $n_{i}=n_{i↑}+n_{i↓}$. The Hamiltonian (5) represents the Hubbard model, which includes nearest-neighbor hopping and on-site interaction, with the addition of a soft-shoulder interaction term with a range of $r_{c}$ sites. In particular, we refer to U,V for the on-site and off-site interaction strengths, respectively. As the Hamiltonian (5) commutes with the total number of particles $N=N_{↑}+N_{↓}$, $N_{\sigma }=\sum_{i}n_{i,\sigma }$, the nature of the ground state is also dictated by the filling; this is defined as $\rho =\rho_{↑}+\rho_{↓}$, with $\rho_{\sigma }=N_{\sigma }/2L$.

#### 3.2.1. $r_{c}=1$ Model

**Figure 3:**
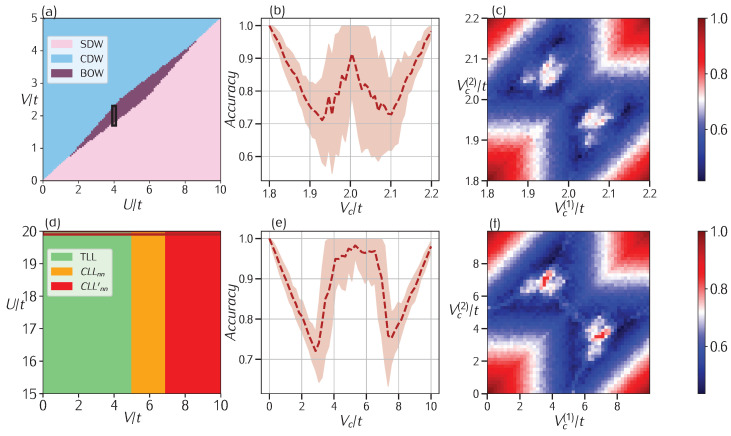
**Two-phase and three-phase learning applied to Extended Hubbard with** $r_{c}=1,2$. (**a**) Phase diagram of the $r_{c}=1$ model showing the CDW and SDW sectors separated by the thin BOW phase. The black rectangle indicates the points where confusion learning was applied. (**b**) For this model, 2-phase learning detects a single phase transition at V=2t, while (**c**) 3-phase learning shows a peak at the two close phase transition points $V_{c}^{\left(1\right)}≃1.9t$ and $V_{c}^{\left(2\right)}≃2.1t$. (**d**) Phase diagram for high *U* values of the $r_{c}=2$ model with three phases; the region investigated with confusion learning is highlighted by the red rectangle. In this case, (**e**) 2-phase learning returns a plateau of high accuracy for all the values inside the $CLL_{nn}$ phase, while (**f**) 3-phase learning shows a clear peak in accuracy at coordinates $V_{c}^{\left(1\right)}≃3.8t$, $V_{c}^{\left(2\right)}≃6.8t$.

Taking $r_{c}=1$ [50] at half-filling $\rho_{↑}=\rho_{↓}=1/4$, i.e., one particle per site, one obtains a relatively simple phase diagram, as shown in Figure 3a. Here, between the usual Charge and Spin Density Wave phases (CDW, light blue/SDW, pink), which are classically separated by the phase transition line for U/t=2V/t, there is a small region exhibiting a Bond-Order Wave (BOW, purple) phase. In particular, this should be present near the classical phase transition line and for V/t∈[1,4] [50]. To test the effectiveness of our method, we generated a set of data with a fixed value of U/t=4 while sweeping V/t in the interval [1.8,2.2], i.e., in the neighborhood of the phase transition line. Although two-phase learning detects a phase transition precisely at V/t=2 (Figure 3b), three-phase learning (Figure 3c) detects the possibility of two phase transitions occurring at values of $V_{c}^{\left(1\right)}≃1.9t$ and $V_{c}^{\left(2\right)}≃2.1t$. This is in agreement with the results present in the literature as these transitions are predicted to occur in the thermodynamic limit and for U=4t at $V_{c}^{\left(1\right)}≃1.877t$ and $V_{c}^{\left(2\right)}=2.164t$. Identifying the two phase transitions is exceptionally challenging due to the narrowness of the BOW phase; in fact, this phase diagram has been the subject of extensive analytical [51,52,53,54,55,56,57] and numerical [58,59,60,61,62] studies in the past. This difficulty explains why even the two-phase scheme produces good results, as evidenced by the W-shape in the accuracy plot. However, it is important to emphasize the effectiveness of our three-phase scheme, which successfully pinpoints the correct phase transition points.

#### 3.2.2. $r_{c}=2$ Model

We now turn our attention to another extension of the model with the same Hamiltonian as Equation (5) but with $r_{c}=2$ and filling ρ=2/5. In this case, we consider a chain of L=30 sites with PBC at filling ρ=2/5, with $\rho_{↑}=\rho_{↓}=1/5$. The choice of L=30 is connected to the frustration of the model for these particular values of $r_{c}$ and ρ. A full description of the phase diagram of this model is far beyond the purposes of the current study; we direct the interested reader to [6,63,64], where the semiclassical large-*U* limit of the model was also studied, yielding the phase diagram shown in Figure 3d. Also, as we consider the strong on-site interaction limit U≫t (in particular, we take U/t=20), the ground state of the model should be similar to the one studied in [65,66] for a spinless chain. In particular, for the same filling ρ=2/5, it was predicted in [66] that a phase should emerge for V∈[4t,6t], separating a Tomonaga Luttinger Liquid phase to a Cluster Luttinger Liquid one. By sweeping V/t∈[0,10], we find that, while the two-phase learning scheme does not display the desired W-shaped accuracy, the three-phase construction presents a peak for $V_{c}^{\left(1\right)}≃3.8t$, $V_{c}^{\left(2\right)}≃6.8t$, values that show a slight offset with respect to the previously mentioned values, possibly because of the CNN we have employed.

## 4. Conclusions

In this work, we proposed a generalization of the Learning by Confusion scheme, a technique that has proved useful in inspecting quantum many-body equilibrium phase diagrams in an unbiased way. While the original scheme was mostly designed to address systems exhibiting only one phase transition point, we tackled the problem of phase diagram regions with three phases. Our construction simply addresses the problem by modifying the underlying Convolutional Neural Network from a binary classifier to a ternary one, enabling the extension to systems with an arbitrary number of phases. Moreover, we tested this construction on a variety of different models, ranging from the integrable case of the free Kitaev chain and its non-integrable interacting extension to the one-dimensional Extended Hubbard model with soft-shoulder potential. Despite the different natures of transition and phases displayed by the aforementioned models, the three-phase confusion approach was always able to identify phase transition points, which is consistent with the existing results in the literature. In addition to the immediate proof of principle regarding the technique itself, our work paves the way to extending Learning by Confusion to a variety of new systems, demonstrating once more the usefulness of Neural Networks within the context of quantum many-body theory.

## Data Availability

The data is available upon request from the authors.

## Abbreviations

The following abbreviations are used in this manuscript:

PBC	Periodic Boundary Condition
DMRG	Density Matrix Renormalization Group
CNN	Convolutional Neural Network

## Appendix A. Data and CNN Details

For each model, we produced a set of observables at different points of the phase diagram to create a dataset. Here, we list the specifics for each model.

### Appendix A.1. Kitaev Chain Data

We define the Fourier transform of the single-particle standard (c(k)) and anomalous (f(k)) correlation functions:

(A1) $$\begin{array}{c} c\left(k\right)=\sum_{i,j}e^{ik(i-j)}\langle a_{i}^{†}a_{j}\rangle \end{array}$$

(A2) $$\begin{array}{c} f\left(k\right)=\sum_{i,j}e^{ik(i-j)}\langle a_{i}a_{j}\rangle \end{array}$$

where the expectation values are taken over the ground state. Notice that c(k) is real, whereas f(k) is purely imaginary. This is due to the antisymmetry of the expectation value $\langle a_{i}a_{j}\rangle$ for the exchange i↔j. For this reason, we only take the imaginary part of f(k). In our non–interacting model (Equation (1)), the correlators c(k) and f(k) can be computed analytically and take the form

(A3) $$\begin{array}{c} c\left(k\right)=\frac{1}{2}+\frac{\mu /2+J\cos k}{2E(k)}, \end{array}$$

(A4) $$\begin{array}{c} f\left(k\right)=\frac{\Delta \sin k}{2E(k)}, \end{array}$$

with E(k) being the energy dispersion relation (2) in the text. We created a dataset of 1500 points with Δ=1 fixed and varying μ in the range [−8,8], making sure to cover each phase transition. Each point is a 2×L matrix with the two correlators stacked to be fed into the Convolutional Neural Network. Then, the dataset was divided into 50 ordered subsets according to their Δ to perform the confusion scheme.

In the interacting case, it is not possible to exactly evaluate the correlation functions c(k) (Equation (A1)) and f(k) (Equation (A2)) on the ground state of the Hamiltonian of Equation (4). Therefore, we calculated them using the DMRG algorithm for a lattice of size L=100. We generated 4000 points for parameters V∈[−4,4] and μ∈[0,5], the phase diagram being symmetrical regarding the transformation μ→−μ. Each point is a 2×100 matrix, the first row being the correlator c(k) (Equation (A1)) and the second being f(k) (Equation (A2)). In the same way as the Kitaev chain, we created a dataset of all the points with μ=3, ordered them according to their *V*, and divided them into 50 subgroups to perform the confusion scheme.

### Appendix A.2. Hubbard Model

In the case of the one-dimensional Extended Hubbard model with soft-shoulder potential with $r_{c}=1$ at half-filling, previous works have shown the presence of three phases for U=4t and V∈[1.8t,2.2t]. These are, for increasing values of *V*, Charge Density Wave, Bond-Order Wave, and Spin Density Wave. For this reason, we identified three observables that are capable of detecting these phases. For CDW and SDW, we take the charge and spin structure factors, defined as

(A5) $$\begin{array}{cc} \; & S_{c}\left(k\right)=\frac{1}{N}\sum_{l,j}e^{-ik(j-l)}\left(\langle n_{l}n_{j}\rangle -\langle n_{l}\rangle \langle n_{j}\rangle \right) \end{array}$$

(A6) $$\begin{array}{cc} \; & S_{s}\left(k\right)=\frac{1}{N}\sum_{l,j}e^{-ik(j-l)}\left(\langle S_{l}^{z}S_{j}^{z}\rangle -\langle S_{l}^{z}\rangle \langle S_{j}^{z}\rangle \right) \end{array}$$

and the BOW order parameter, defined as

(A7) $$B_{i}=\langle c_{i}^{†}c_{i+1}+c_{i+1}^{†}c_{i}\rangle .$$

Observables Equations (A5) and (A6) have length L/2, whereas Equation (A7) has length *L*. Therefore, the observables for each data point were stacked into a 2×L matrix.

For the one-dimensional Extended Hubbard model with soft-shoulder potential with range $r_{c}=2$ at filling ρ=2/5, we consider the same charge and spin structure factors as defined in Equations (A5) and (A6) and the local density, defined as

(A8) $$n(x_{i})=\langle n_{i}\rangle .$$

Once again, the observables were arranged into a 2×L matrix to be fed into the CNN.

### Appendix A.3. CNN

The Convolutional Neural Network used in this work accepts as input 2×L matrix points composed of stacked observables, as explained in the previous sections. The data undergo one 2D convolutional layer with 5 filters of kernel size 2, a 1D convolutional layer with 5 filters and kernel size 1, followed by a ReLU activation function. Afterwards, there is one linear layer with sigmoid activation function and n=2(3) output neurons if the 2(3)-phase learning scheme is adopted.
